# In-vitro effects of novel periodontal scalers with a planar ultrasonic piezoelectric transducer on periodontal biofilm removal, dentine surface roughness, and periodontal ligament fibroblasts adhesion

**DOI:** 10.1007/s00784-024-05671-w

**Published:** 2024-05-03

**Authors:** Luciana Aranha Berto, Johanna Blanda Ettmayer, Diego Stutzer, Sandor Nietzsche, Thomas Niederhauser, Juergen Burger, Anton Sculean, Sigrun Eick, Martin Hofmann

**Affiliations:** 1 Department of Periodontology, School of Dental Medicine, University of Bern, Freiburgstrasse 7, Bern, CH-3010 Switzerland; 2 School of Biomedical and Precision Engineering, University of Bern, Gueterstrasse 24/26, Bern, CH-3008 Switzerland; 3 Institute for Human Centered Engineering, Bern University of Applied Sciences, Quellgasse 21, Biel, CH-2501 Switzerland; 4 Center of Electron Microscopy, University Hospital Jena, Ziegelmühlenweg 1, D-07743 Jena, Germany

**Keywords:** Periodontal therapy, Biofilm removal, Surface roughness, Piezoelectric ultrasonic scaler

## Abstract

**Objectives:**

To compare ultrasonic scaler prototypes based on a planar piezoelectric transducer with different working frequencies featuring a titanium (Ti-20, Ti-28, and Ti-40) or stainless steel (SS-28) instrument, with a commercially available scaler (com-29) in terms of biofilm removal and reformation, dentine surface roughness and adhesion of periodontal fibroblasts.

**Materials and methods:**

A periodontal multi-species biofilm was formed on specimens with dentine slices. Thereafter specimens were instrumented with scalers in a periodontal pocket model or left untreated (control). The remaining biofilms were quantified and allowed to reform on instrumented dentine slices. In addition, fibroblasts were seeded for attachment evaluation after 72 h of incubation. Dentine surface roughness was analyzed before and after instrumentation.

**Results:**

All tested instruments reduced the colony-forming unit (cfu) counts by about 3 to 4 log_10_ and the biofilm quantity (each *p* < 0.01 vs. control), but with no statistically significant difference between the instrumented groups. After 24-hour biofilm reformation, no differences in cfu counts were observed between any groups, but the biofilm quantity was about 50% in all instrumented groups compared to the control. The attachment of fibroblasts on instrumented dentine was significantly higher than on untreated dentine (*p* < 0.05), with the exception of Ti-20. The dentine surface roughness was not affected by any instrumentation.

**Conclusions:**

The planar piezoelectric scaler prototypes are able to efficiently remove biofilm without dentine surface alterations, regardless of the operating frequency or instrument material.

**Clinical relevance:**

Ultrasonic scalers based on a planar piezoelectric transducer might be an alternative to currently available ultrasonic scalers.

## Introduction

Periodontitis is an inflammatory disease which affects the periodontal tissue around teeth. Its pathogenesis results from an interaction of a dysbiotic microbiota with a dysregulated host response [1]. Keystone bacteria such as *Porphyromonas gingivalis* may convert an eubiotic microbiota into a dysbiotic one accompanied by triggering host response and influenced by genetic factors [2]. Patients’ risk factors include diabetes, smoking, diet, and poor oral health care [3]. The dysbiotic microbiota exists within biofilms, a highly organized community with different behavior of the microorganisms compared to free-floating ones and surrounded by a biofilm matrix [4]. The biofilm matrix not only protects the microorganisms against antimicrobials and immune response, but also provides stability and resistance to mechanical removal [5]. Nowadays, the dental plaque biofilm is even claimed as a host tissue, since it normally contributes to the maintenance of health, but can also lead to disease when disrupted [6].

Activity of antimicrobials against bacteria in biofilms is extremely limited not only by the development of antimicrobial resistance per se, but also by the inability of the antimicrobials to penetrate through the biofilm matrix, a modified chemical environment and subpopulations of microbes in the biofilms [7]. Therefore, the gold standard in the control of biofilm-associated diseases is still the mechanical removal of biofilms. In the case of periodontitis, the S3 level clinical practice guideline of the European Federation of Periodontology (EFP) underlines the importance of oral hygiene measures and recommends the professional supragingival dental biofilm control in the first step of periodontal therapy [8]. In the second step of therapy, subgingival instrumentation is recommended aiming to reduce probing pocket depths (PPD), gingival inflammation, and, thus, the number of diseased sites [8]. The instrumentation can be performed with hand instruments and / or power-driven sonic or ultrasonic instruments [8]. A survey in Europe found that powered and hand scalers are used to the same extent in this step of therapy, as 94% of the about 2000 responding periodontists and dental hygienists used a combination of both instrument types [9]. Further, professional supragingival dental biofilm control is recommended as part of supportive periodontal care and should be performed at time intervals of 3–12 months [8]. Periodontal therapy and continuous supportive periodontal care are essential in preventing tooth loss [10].

Powered scalers have been introduced in periodontal therapy in the mid of the last century. The instrument tips are operated at sonic (6–8 kHz) or ultrasonic frequencies (25–42 kHz), with the most common frequency for commercial ultrasonic scalers being 25–30 kHz [11, 12]. Ultrasonic scalers generate the vibrations using either a piezoelectric or a magnetostrictive material subjected to an alternating electric or magnetic field, respectively [11]. Meanwhile, there is consensus that manual hand instruments and powered scalers are both suitable for periodontal therapy [13, 14]. Also, when using after periodontal surgical therapy, a recent systematic review did not find a difference regarding PPD reduction between hand and power-driven instruments [15]. In addition, piezoelectric scalers were equally accepted by patients as magnetostrictive ones in terms of pain, discomfort, noise, and vibration sensation when used with water irrigation at room temperature [16]. Technically a scaler must meet certain requirements. According to the International Organization for Standardization (ISO) 18397:2016, electric powered scalers must operate in a frequency range between 18 kHz and 60 kHz, incorporate a tip made of biocompatible material, and withstand > 250 reprocessing cycles. In addition, the scalers shall not exceed a vibration amplitude of 200 μm (peak-peak).

In the present study, prototypes of a novel planar piezoelectric ultrasonic scaler were tested in comparison with a commercial piezoelectric scaler in a periodontal pocket model. The monolithic planar concept, in which the ultrasonic transducer and instrument tip are permanently connected, was first introduced for ultrasonic surgery [17–19]. This concept provides high vibration transmission, allows weight reduction, and simplifies production. The concept has been refined and adapted for periodontal therapy, and technical details of the development and characteristics of the new scalers have recently been published [20]. The developed planar ultrasonic scalers are adaptable to different resonant frequencies and, thus, allow the influence of the operation frequency to be investigated. For this purpose, prototypes with a nominal operating frequency of 20 kHz, 28 kHz, and 40 kHz were designed and manufactured. In addition to 28 kHz, which allows for comparison to a commercially available scaler and, thus, corresponds to the most common frequencies for ultrasonic scalers, two further frequencies were selected with a deviation of ± 10 kHz. As the development of the planar scalers was derived from titanium-based planar scalpels [17], the same titanium alloy grade 5 was used for the scaler instrument. Additionally, a prototype with a stainless steel (316 L) instrument operating at a frequency of 28 kHz was manufactured and tested in the present study for comparison purposes.

The questions to be answered in the present study were:

i) Are the new prototypes able to remove biofilm as efficient as the commercial scaler?
ii) Is the application of the new prototypes able to inhibit reformation of biofilm?
iii) Is the application of new prototypes able to promote reattachment of periodontal ligament fibroblasts?
iv) In the in-vitro assays, prototypes generating three different frequencies were applied. Is it possible to use frequencies outside the commonly used range (25–30 kHz) for biofilm removal?
v) Is a planar piezoelectric scaler based on titanium equally effective in removing biofilm as one with the same design and operating frequency (28 kHz) but made of stainless steel?

## Materials and methods

### Tested instruments

**Fig. 1:**
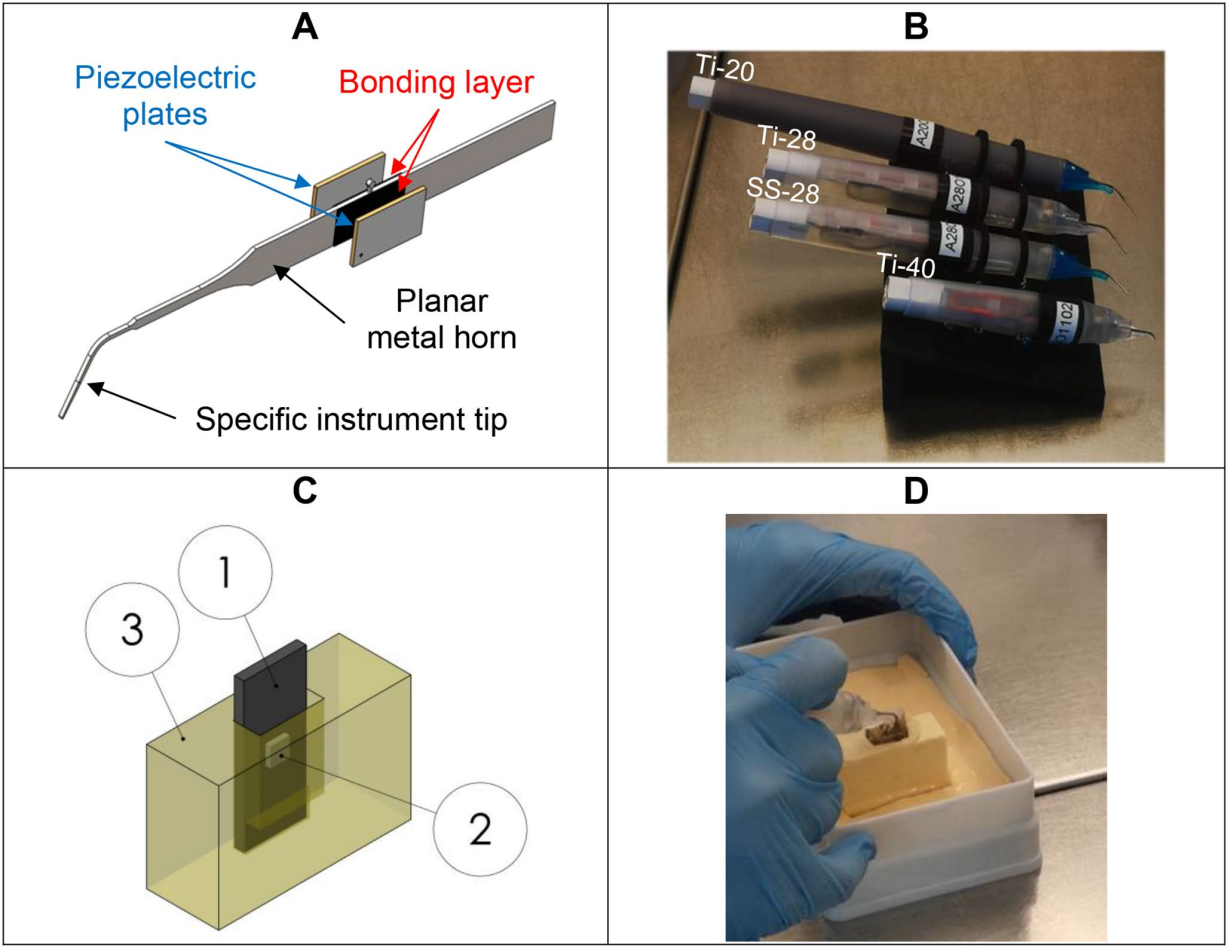
Concept of the planar scaler (**A**), photograph of the different prototypes that were tested (**B**), and illustration of the periodontal pocket model (**C**) with the plastic specimen (1) and attached dentine slice (2) placed in the artificial pocket (3). Instrumentation for 10 s (**D**)

The novel scalers are based on a planar design with two transversely vibrating piezoelectric plate actuators adhesively bonded to both sides of a planar horn (Fig. 1A). The instruments that were tested are shown in Fig. 1B and comprised titanium prototypes operating at nominal frequencies of 20 kHz (Ti-20), 28 kHz (Ti-28), and 40 kHz (Ti-40), and a stainless steel prototype operating at 28 kHz (SS-28). They were compared to a commercially available ultrasonic scaler (AIRFLOW® PROPHYLAXIS MASTER with PIEZON® handpiece and PS instrument, Electro Medical Systems S.A., Nyon, Switzerland) operating at 29 kHz (com-29). To investigate the influence of the operating frequencies, an electronic control system for driving the prototypes was adjusted such that a nominal displacement of about 40 μm (peak-to-peak) was achieved at the extremity of the instrument tip, independent of the frequency. This displacement amplitude is comparable to the PIEZON® system’s power level 3 recommended for biofilm removal and used in the experiment.

### Specimens

The preparation of the test specimens was carried out according to a previous study [21]. In short, dentine slices with a standardized surface and a size of about 4 mm x 4 mm x 2 mm were prepared from extracted teeth. Prior to the extraction, patients were informed about the use of their teeth, and cells attaching to teeth such as periodontal ligament (PDL) fibroblasts, for research purposes and their written informed consent was obtained. This procedure is in accordance with the approved guidelines and regulations of the local Cantonal Ethics Committee of Bern (KEK) for irreversibly anonymized samples. The dentine slices were fixed on plastic (polycarbonate) pieces with a size of about 10 mm x 30 mm. The prepared specimens were kept in 0.9% w/v NaCl solution. Immediately before the experiments, both sides of the specimen were irradiated with UV for 30 min.

### Biofilm formation

The recent biofilm protocol used for the pocket model [21] was slightly modified. Modifications were made regarding the composition, the coating solution, and the incubation of the tubes before placing in the pocket model.

In the present study, the 12-species periodontal biofilm, consisting of *Streptococcus gordonii* ATCC 10,558, *Actinomyces naeslundii* ATCC 12,104, *Fusobacterium nucleatum* ATCC 25,586, *Campylobacter rectus* ATCC 33,238, *Capnocytophaga gingivalis ATCC 33,624*, *Eikenella corrodens* ATCC 23,834, *Parvimonas micra* ATCC 33,270, *Prevotella intermedia* ATCC 25,611, *Porphyromonas gingivalis* ATCC 33,277, *Tannerella forsythia* ATCC 43,037, *Treponema denticola* ATCC 35,405, *Filifactor alocis* ATCC 33,099 [22], was formed on the surface of the dentine specimens.

First, the dentine slices were coated with a 1.5% bovine serum albumin (BSA) and 0.27% mucin solution for 30 min. The specimens were placed in tubes. Bacteria were suspended in 0.9% w/v NaCl according to McFarland 4, mixed (one part *S. gordonii*, two parts *A. naeslundii* and four parts of each of the other species), and diluted 1:9 with Wilkins-Chalgren broth (Oxoid, Basingstoke, UK), supplemented with 5% lysed sheep blood, 400 µg/ml niacinamide, 1 mg/ml cysteine, and 5 µg/ml cocarboxylase. This mixture was added to the tubes which were incubated for 4.5 days with renewed addition of *P. gingivalis*, *T. forsythia* and *T. denticola* at the third day of incubation.

### Periodontal pocket model and instrumentation

A previously developed periodontal pocket model [21, 23] to mimic the clinical situation of a subgingival root scaling procedure was further improved. A polysiloxane precision impression material (Optosil, Kulzer, Hanau, Germany) was applied to a polylactic acid (PLA) negative mold that was 3D printed (Creator 3 Pro, Flashforge, Zhejiang, China) using a fused deposition modeling (FDM) technology (Fig. 1C). The shape of the artificial pocket ensured a stable position of the specimen during instrumentation and maintaining a well-defined narrow distance between the specimen and the wall of the pocket wall that corresponds to clinical conditions. When placed inside the pocket model (Fig. 1C), the upper and lower edges of the dentine slices were always in the same position, 2 and 6 mm below the silicone surface, respectively.

After 4.5 days of biofilm formation, specimens were treated by mechanical debridement in the artificial pocket model using one of the five instruments (Fig. 1D). All treatments were performed by the same well-trained and calibrated periodontist (J.B.E.), with slow tip movements (approximately 1 stroke/s) for 10 s in a perpendicular direction. During instrumentation, irrigation with sterile water at a flow of 75 ± 9 ml/min was applied.

**Fig. 2:**
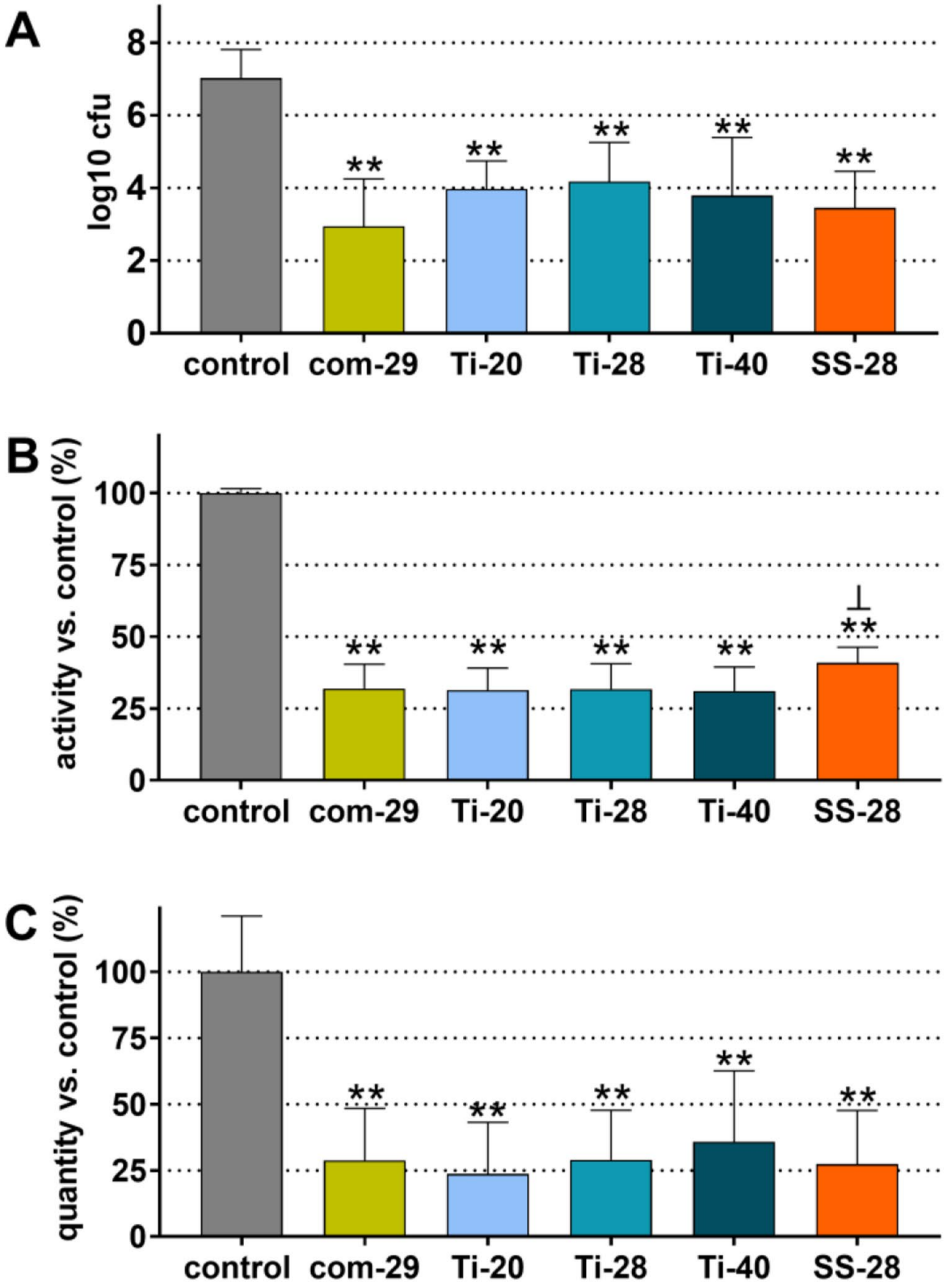
Colony-forming units (cfu; **A**), metabolic activity (**B**) and quantity (**C**) of the remained biofilm after instrumentation with a commercial ultrasonic scaler (com-29) or different prototypes (Ti-20, Ti-28, Ti-40, SS-28). ** *p* < 0.01 vs. control, ┴ *p* < 0.05 vs. each other instruments (com-29, Ti-20, Ti-28, Ti-40)

**Fig. 3:**
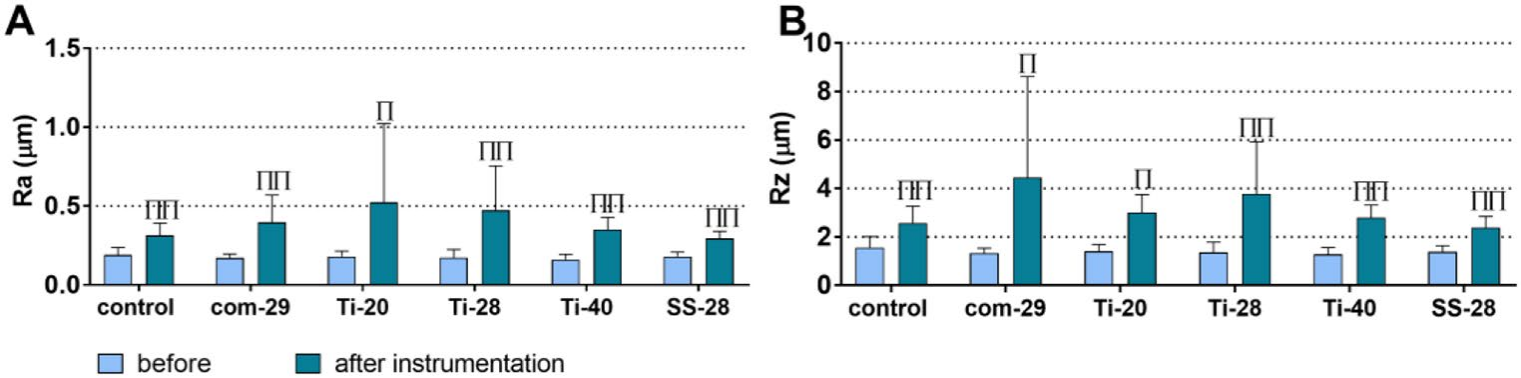
Surface roughness (Ra (**A**) and Rz (**B**) values) before and after treatments with the commercial ultrasonic scaler (com-29) or different prototypes (Ti-20, Ti-28, Ti-40, SS-28). ^Π/ΠΠ^*p* < 0.05/*p* < 0.01 after vs. before instrumentation

**Fig. 4:**
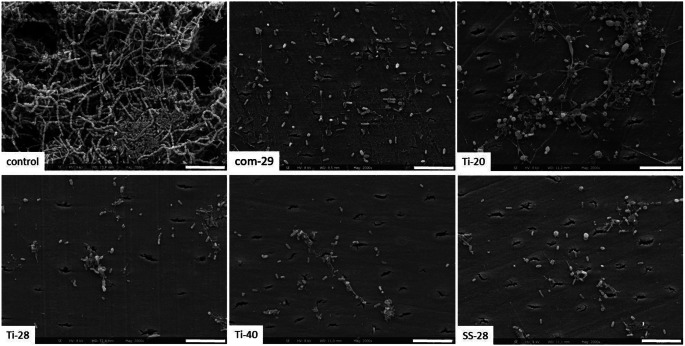
Scanning electron microscopy photographs of the dentine surface after biofilm formation and instrumentation (commercial ultrasonic scaler (com-29) or different prototypes (Ti-20, Ti-28, Ti-40, SS-28)). Scale bar: 10 μm

**Fig. 5:**
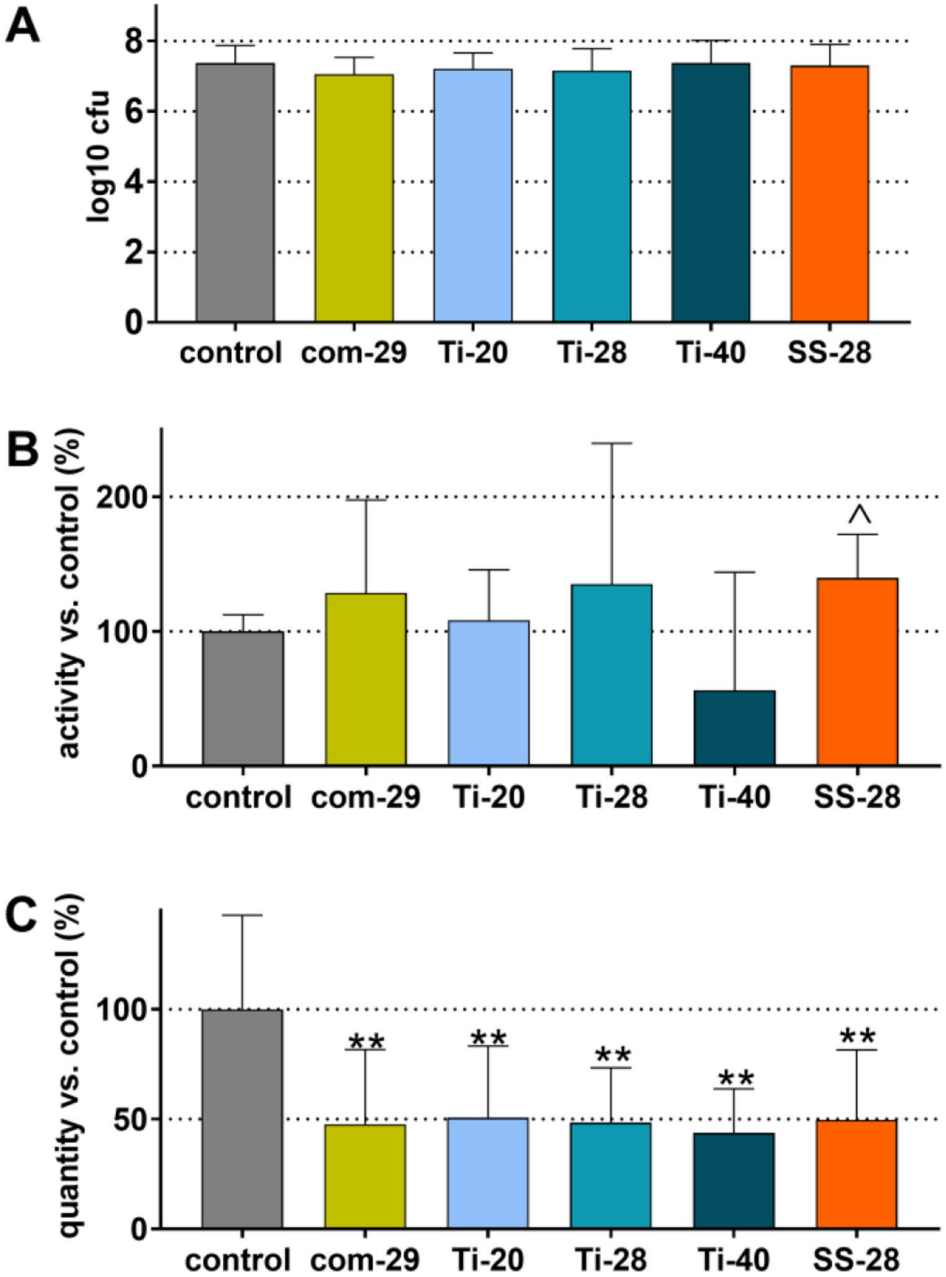
Colony-forming units (cfu; **A**), metabolic activity (**B**) and quantity (**C**) of the reformed biofilm after instrumentation with a commercial ultrasonic scaler (com-29) or different prototypes (Ti-20, Ti-28, Ti-40, SS-28). ** *p* < 0.01 vs. control, ┴ *p* < 0.05 vs. each other instruments (com-29, Ti-20, Ti-28, Ti-40)

**Fig. 6:**
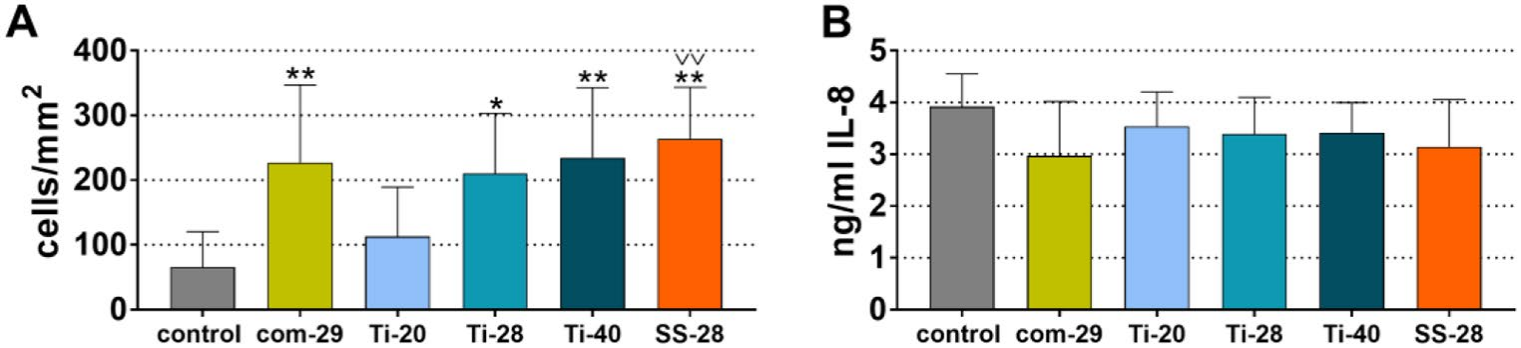
Attached PDL fibroblasts (**A**) to instrumented dentine specimens 72 h after incubation and IL-8 levels (**B**) in the cell culture media after 40 h of incubation.*^/^** *p* < 0.05/*p* < 0.01 vs. control, ^**˅˅**^*p* < 0.01 vs. Ti-20

### Quantification of remaining biofilm

Following treatments, the biofilm was collected from the dentine surface by intensive wiping with a cotton swab and suspended in 0.9% w/v NaCl solution. After serial dilution, 25 µl of each suspension were plated onto tryptic soy agar (Oxoid) plates with 5% sheep blood. The TSA plates were incubated in anaerobic conditions for 10 d. Thereafter, the total counts of colony-forming units (cfu) were recorded [21]. In addition, the remaining biofilm was quantified by means of crystal violet staining, and the metabolic activity was assessed using Alamar blue as a redox indicator [24–26].

### Recolonization after treatments

To analyze biofilm reformation, treated and untreated specimens were exposed to UV for 30 min to inactivate potential contaminants, as the treatment could not be performed in a sterile manner. The dentine slices were coated again with a 1.5% BSA and 0.27% mucin solution for 30 min before they were placed in new tubes for a renewed biofilm formation. After 24 h of incubation, the biofilm was again analyzed as described before (total bacterial counts, metabolic activity, biofilm quantity).

### Measurement of surface roughness

Dentine surface roughness was analyzed with an optical profilometer (FRT MicroProf® 100, equipped with an H0 sensor, Fries Research & Technology GmbH, Bergisch Gladbach, Germany) before and after the treatments. Six linear traces of 2 mm were recorded at a pixel density of 1000/mm. The average surface roughness (Ra) and the arithmetic mean height of the surface profile (Rz) were then determined for all traces using the Mark III software (Fries Research & Technology GmbH) [27].

### Attachment of periodontal ligament fibroblasts and release of IL-8

A protocol in accordance with previously conducted experiments [21] was applied. Human PDL fibroblasts harvested anonymously from extracted teeth were cultivated in DMEM media (Life Technologies / Invitrogen, Paisley, UK) with 10% fetal calf serum (FCS; Life Technologies / Invitrogen), incubated at 37 °C with 5% CO_2_, and used always in the fourth passage. After treatments, the dentine slices were removed from the plastic holder, placed into 48-well plates and exposed to UV for 30 min. Thereafter, PDL fibroblasts in DMEM with 10% FCS were added at a density of 10,000 cells/well. After 40 h, the culture medium was exchanged, and the spent media was used to quantify released interleukin (IL)-8 using a commercially available enzyme-linked immunosorbent assay (ELISA) kit (R&D Systems Europe Ltd., Abingdon, UK), according to the manufacturer’s instruction. After 72 h of incubation, the fibroblasts were fixed and stained with Hemacolor® (Merck KGaA, Darmstadt, Germany). The attached fibroblasts were quantified by using a fluorescent microscope (Olympus BX51, Tokyo, Japan). For each specimen, the number of fibroblasts in three fields of 1 mm^2^ per sample was counted and the mean value was used for evaluation.

### Scanning electron microscopy (SEM)

Scanning electron microscopy (SEM) was applied to visualize surface structure and biofilm removal. Dentine slices were fixed with 2% glutaraldehyde in cacodylate buffer for 30 min after instrumentation. Subsequently, they were washed with cacodylate buffer and dehydrated. Following critical point drying, the samples were sputter-coated with gold and examined with a ZEISS LEO-1530 Gemini (Carl Zeiss NTS GmbH, Oberkochen, Germany) equipped with a field emission electron gun operating at 10 keV.

### Statistical analysis

Data are presented as mean and standard deviation (SD). In case of bacterial loads (total cfu counts) data were transferred to log_10_ values. All results were compared using one-way analysis of variance (ANOVA), followed by Bonferroni post-hoc test for multiple comparisons of groups. Surface roughness values before and after instrumentation were analyzed by a paired t-test. A p-value of 0.05 was considered statistically significant. SPSS software (version 28.0, IBM, Chicago, IL, USA) was used for statistical analysis. A minimum of 10 samples was evaluated per group for each analysis after five independent experiments.

## Results

### Biofilm removal

After treatments, viable bacterial counts were determined as cfu after anaerobic cultivation on agar plates. Figure 2A shows the cfu counts of the remaining biofilm expressed in log_10_. In the absence of treatment (control group), the biofilm contained in mean 7.02 ± 0.79 log_10_ cfu. All instruments were capable of statistically significantly reducing the cfu counts in the biofilm on the dentine surface compared to the control group (*p* < 0.001). The lowest reduction was 2.85 log_10_ (Ti-28) and the highest was 4.08 log_10_ (Com-29). However, when comparing the performance of the five tested devices in terms of cfu reduction, no statistically significant differences were found (*p* > 0.05). The results of measuring metabolic activity in the biofilms (Fig. 2B) and quantifying the biofilms by crystal violet staining (Fig. 2C) were similar. All devices were able to reduce biofilm metabolic activity (by 30–59%; each *p* < 0.001) and quantity (by 72–76%; each *p* < 0.001) on treated samples compared to the control group. However, no significant differences were found between the tested devices in terms of biofilm quantity (*p* > 0.05). Regarding metabolic activity, higher values were measured after treatment with SS-28 compared to each other instrument (*p* = 0.012-*p* = 0.031).

### Alterations in surface roughness after treatment

Prior to instrumentation, no significant differences in surface parameters were found between the groups. The mean Ra (Fig. 3A) and Rz (Fig. 3B) values ranged from 0.16 to 0.19 μm and 1.28–1.56 μm, respectively. After treatment, the mean Ra and Rz values increased to 0.30–0.52 μm and 2.39–4.46 μm. The increase of the Ra and Rz values was statistically significant in all groups. Regarding Ra and Rz values after treatments and the intragroup differences before and after treatments, there were no statistically significant differences between the groups.

### Scanning electron microscopy photographs

The SEM photographs confirmed the other findings on the biofilm removal. After instrumentation, only a few remnants remained on the dentine surface, with no clear difference between the instrument groups. There might be a trend towards less bacteria after instrumentation with Ti-40 than with Ti-20. Further, no obvious damages to the dentine surface were observed (Fig. 4).

### Biofilm reformation

After 24 h of biofilm reformation on the dentine slides, the mean bacterial counts ranged from 7.05 log_10_ cfu (com-29) and 7.37 log_10_ cfu (both control, Ti-40) with no statistically significant difference between the groups (*p* > 0.05; Fig. 5A). Similarly, there were no statistically significant differences between any instrumentation and the control group in terms of biofilm metabolic activity. Comparison among the instrumentation groups revealed a higher activity after treatment with SS-28 vs. Ti-40 (*p* = 0.036; Fig. 5B). The biofilm quantity was significantly higher in the control group than in the other groups (about 50% of the control group each, *p* < 0.001), with all treatment groups having similar biofilm quantity after recolonization (*p* > 0.05; Fig. 5C).

### Attachment of periodontal ligament fibroblasts and released IL-8

Attachment of PDL fibroblasts to instrumented dentine slices was significantly higher for all instrumented groups when compared to the control group, with the exception of Ti-20 (Fig. 6A). The highest number of attached PDL fibroblasts was counted after instrumentation with SS-28 (*p* < 0.001; vs. control), followed by Ti-40 and com-29 (*p* = 0.002, *p* = 0.003; vs. control). Among the instrumented groups, the only significant difference was found between Ti-20 and SS-28 (*p* < 0.001). The IL-8 levels (Fig. 6B) in the cell culture media after 40 h of incubation were not significantly different between groups (*p* > 0.05).

## Discussion

In the present in-vitro study, the potential of ultrasonic scalers based on a planar transducer concept was evaluated using a biofilm periodontal pocket model. The comparison with a widely used commercial scaler revealed that there were no highly significant differences between the prototypes and the commercial scaler in terms of biofilm removal, surface damage, biofilm recolonization, and PDL fibroblast attachment to the instrumented surface.

The used periodontal pocket model allows realistic instrumentation by imitating the narrow gap between tooth and periodontium and guiding the application of the scaler’s instrument tip perpendicular to the dentine surface. The orientation of the tip has been reported to affect biofilm removal [28], which underlines the need to correctly use the scaler also under in-vitro conditions. However, a standardized dentine slice with a flat surface does not completely correspond to reality, since the dentine is not covered by cementum, and the preparation of the slices creates open dentine tubules. Consequently, the damage to the root cementum and the behavior of the instruments on tooth curvatures and furcations could not be studied, which might be a limitation of the model.

All instruments removed the biofilm to a similar extent and no significant difference was found between them. However, the commercial scaler (com-29) tended to be more effective in reducing cfu counts. The cfu reduction after instrumentation was similar to that of an ultrasonic scaler in a previous study [21]. It is also worth noting that in the aforementioned study, ultrasonication showed the highest effectiveness in reducing biofilm with statistically significant differences to instrumentations with hand curette or air-polishing [21].

Overall, the planar scalers were found to have no additional potential for hard tissue surface damage. A minor increase of the surface roughness was consistently observed after treatment vs. untreated. However, since this increase was also found in the control samples, it might be caused by the biofilm-induced demineralization and not by the applied instrumentation. Our results, indicating no surface damage, may confirm a few other in-vitro studies that have found less surface roughness after applying piezoelectric scalers compared with manual scaling [21, 29]. However, contrasting results have also been reported, with a hand curette producing smoother surfaces without grooves compared to ultrasonic scaling and air-polishing [30].

Reformation of biofilm on the treated surfaces did not result in any significant differences regarding the cfu counts, but the biofilm quantity was about 50% when compared to the control. These results underline the efficient removal of the biofilm by the instrumentation, but they are also indicative of less viable bacteria in older biofilms. The stability of cfu counts in a mature biofilm was demonstrated in ex-vivo experiments [31]. Furthermore, a clear increase in biofilm quantity was observed on test specimens worn in the oral cavity for up to 3 days, with no difference over time when live/dead staining was applied [32]. However, live/dead staining based on membrane integrity is not an equivalent for the ability of bacteria to form colonies [33].

Removing biofilms supports the adhesion of PDL fibroblasts. Our recent in-vitro study showed that the adhesion of fibroblasts negatively correlated with the presence of biofilm; the lowest numbers of attached fibroblasts were counted in the untreated biofilm and in the manual scaling groups, whereas the highest attachment was found after ultrasonication [21]. Another study, using extracted and decontaminated teeth (without biofilm remains), found the highest PDL fibroblasts counts after treatment with Er: YAG laser, followed by ultrasonic instrumentation, and the lowest number was quantified on the untreated surface [34]. Although there was no statistically significant difference with respect to remaining biofilm in the present study, PDL fibroblasts attached less to dentine surfaces treated with Ti-20. In addition, SEM photographs after the use of the Ti-20 may indicate more remaining matrix components compared to the other instrumented surfaces. The release of IL-8 from PDL fibroblasts appeared to be inhibited after treatment but with no statistically significant difference. In similar experiments, a statistically significant lower release of IL-8 from PDL-fibroblasts [21] or epithelial cells [23] was found. The result of the present study might be influenced by the low number of attached cells in the controls, which obviously release more IL-8 per cell, and by free-floating fibroblasts in all groups.

The transducers and instrument tips used in the experiments were made of either titanium or stainless steel. Stainless steel, as used for the prototype SS-28 and the commercial scaler (com-29), is the most used material for dental instruments, mainly because of its high strength and hardness and the associated reduced wear [37]. However, tips of conventional stainless steel are not suitable for treatment on titanium implants as they cause scratches on titanium surfaces [35]. Although titanium tips are less commonly used in periodontal therapy, titanium-based prototypes have been developed and included in the study since the design of the planar scaler was derived from the proof of concept with titanium-based planar surgical scalpels [17]. One advantage of titanium tips is their higher elasticity, allowing higher vibration amplitudes and lower hardness, which could be beneficial in treating patients with peri-implantitis by reducing the risk of surface damage to implants. Tips of stainless steel were reported to have a Vickers hardness of about 580 VHN [36], whereas for titanium alloy (grade 5), a Vickers hardness of about 340 VHN was determined [37]. In the present study, the comparison between prototypes with a stainless steel and a titanium instrument operating at the same frequency showed no difference with respect to biofilm removal, reformation of biofilm, surface roughness and PDL fibroblast attachment. These results may indicate the suitability of titanium as a material for ultrasonic instruments used for both periodontal and peri-implant therapy. However, the treatment efficacy on titanium implants might differ from that on dentine [38]. It might be of interest to include not only dentine or titanium discs but also commercial dental implants in future in-vitro experiments to evaluate the performance of the scalers on implant threads.

Most commercial piezoelectric systems operate at a frequency between 25 and 30 kHz [11, 12]. However, there is little evidence that this frequency is optimally chosen. For this reason, lower (20 kHz) and higher (40 kHz) operating frequencies were also tested in our study for their effectiveness in removing biofilm. It was found that fewer PDL fibroblasts were attached to surfaces treated with the 20 kHz prototype compared to the other prototypes. On the other hand, an instrument with a higher operating frequency (40 kHz) could be advantageous. By increasing the frequency and maintaining the displacement amplitude, the velocity increases, resulting in a higher cavitation volume. The cavitation phenomena and the associated biophysical effects, such as shock waves and microstreaming, contribute to the disruption of dental biofilm [39]. Furthermore, the length of the device and, thus, the weight can be reduced, improving handling and user comfort. In addition, it was found that a higher frequency results in less audible noise during instrumentation, which is more comfortable for operators and patients.

This study focused on biofilm removal on defined surfaces. In the experiments using the periodontal pocket model, all instruments were able to remove biofilm by at least 3 log_10_ cfu counts compared to the control group. These results confirm the feasibility and effectiveness of ultrasonic scalers based on the planar concept in biofilm removal. However, this study did not investigate the aspect of dental calculus removal. Titanium tips are believed to also be effective in the removal of calcified deposits. A comparison of stainless steel and titanium hand curettes found only a minor difference in removing artificial calculus on roughened titanium surfaces [40]. However, to the best of our knowledge, no data are available on the comparison of titanium and stainless steel tips in the ultrasonic removal of dental calculus.

In the present study, the power level for all scalers tested was set to achieve a nominal displacement of about 40 μm (peak-to-peak) at the extremity of the instrument tip. This setting corresponds to the power level 3 of the com-29 system, a choice of power that was also made in other studies [30]. It might be of interest to additionally investigate the effects of lower and higher power levels and corresponding amplitudes. In an in-vivo study with hopeless teeth, three different power levels of an ultrasonic scaler were tested regarding the impact on the roughness of dental surfaces. Analysis after extraction found an increased surface roughness vs. control only after using a low power level, but no difference between the different power levels [41].

The main difference between the tested prototypes and conventional ultrasonic scalers is the design concept, in which piezoelectric plates are adhesively bonded to a planar horn with a monolithically integrated tip. The planar design allows for mass reduction, and in combination with the integrated tip, the system provides greater effectiveness and sensitivity, e.g. to detect different load situations. Furthermore, the planar design might also reduce costs and, thus, enable access to new markets. Ecological points but also clinical aspects, such as the treatment of furcations or implant surfaces, raise the question of specific and exchangeable tips in the future.

And finally, besides the variables analyzed under in-vitro conditions, it is necessary to evaluate the performance of the new ultrasonic scalers in clinical settings. In addition to clinical effectiveness, the patients’ and clinicians’ opinions as well as measurements of noise emissions should be investigated. The patients complications should include among others soft and hard tissue damage, pain, and postoperative dentine hypersensitivity [42].

## Conclusion

In conclusion, the prototypes of the planar piezoelectric scalers were able to remove biofilm efficiently without statistically significant difference to a commercial ultrasonic scaler or between configurations with different operating frequencies or instrument materials. It might be of interest to conduct further tests with the prototypes to investigate the influence of different operation frequencies on dental calculus removal, cavitation volume, noise emissions, and clinical effectiveness and acceptance.

## Data Availability

No datasets were generated or analysed during the current study.
